# Clozapine-Induced Refractory Colonic Pseudo-Obstruction

**DOI:** 10.7759/cureus.53377

**Published:** 2024-02-01

**Authors:** Diana Siriwardena, Chahaya M Gauci, Ali Mohtashami, Sarit Badiani, Shahrir Kabir

**Affiliations:** 1 Colorectal Surgery, Royal North Shore Hospital, Sydney, AUS; 2 Medicine and Health, University of Sydney, Sydney, AUS; 3 Medicine and Surgery, University of New South Wales, Sydney, AUS; 4 Surgery, Hepatobiliary and Surgical Oncology Unit, St George Hospital, Kogarah, AUS; 5 Surgery, Bankstown-Lidcombe Hospital, Sydney, AUS

**Keywords:** pseudo-obstruction, colonic pseudo-obstruction, refractory pseudo-obstruction, colectomy, clozapine

## Abstract

The management of treatment-resistant schizophrenia (TRS) is challenging as the medications involved, often atypical antipsychotics, have a host of associated adverse effects. While complications such as agranulocytosis are well established and necessitate close hematological monitoring, the gastrointestinal effects of particular atypical antipsychotics, such as clozapine, are recognized to a lesser extent. The following case of TRS leading to chronic treatment-resistant pseudo-obstruction, eventually requiring total colectomy, highlights the considerable sequelae of clozapine on the gastrointestinal tract. Beyond the effects of severe constipation, the possible implications of ischemic colitis, stercoral perforation, and intraabdominal sepsis warrant a degree of caution when prescribing such medication. This study sheds light on the importance of monitoring bowel motility when administering antipsychotics, particularly clozapine, to avoid these deleterious consequences.

## Introduction

Stercoral colitis can be a life-threatening condition following the use of atypical antipsychotics, particularly clozapine [1]. Clozapine has recognized anticholinergic effects on the gastrointestinal tract, resulting in dysmotility, fecal impaction, increased intraluminal pressure, and risk of intestinal ischemia [2]. Although the current literature explores cases of gastrointestinal hypomotility, stercoral colitis as well as acute colonic ischemia and death, cases of prolonged and pseudo-obstruction refractory to non-operative management, resulting in colonic necrosis from chronic ischemia, that necessitates emergency total colectomy are uncommon. This case highlights the importance of monitoring the prescription of clozapine and considering early surgical intervention in refractory cases to minimize the risk of detrimental outcomes.

## Case presentation

A 60-year-old female with a past medical history significant for treatment-resistant schizophrenia (TRS) presented to the emergency department with fevers, hypotension, and tachycardia. Over the course of her hospital admission, she developed refractory colonic pseudo-obstruction and was found to have chronic ischemic colitis, eventually suffering major hemorrhage and secondary intraabdominal sepsis, such that she required emergency total colectomy and end ileostomy.

Initial symptoms included vomiting, overflow diarrhea, and reduced consciousness (with a Glasgow Coma Score of 13). She had been an inpatient at a mental health hospital for six months prior, being commenced on clozapine during this period. Besides clozapine 450 mg nocte, she was on no other antipsychotic medications. A febrile illness, hypotension, and tachycardia alongside a venous lactate of 10, flagged concerns for possible intraabdominal sepsis and resulted in transfer to a major tertiary hospital.

**Figure 1:**
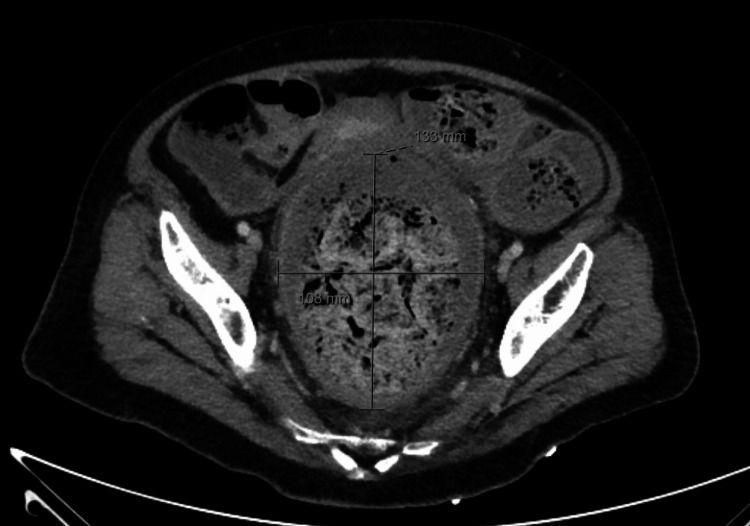
Stercoral colitis with marked distension of the rectum and mild distension of the sigmoid and ascending colon with fecal material. No evidence of perforation was noted.

**Figure 2:**
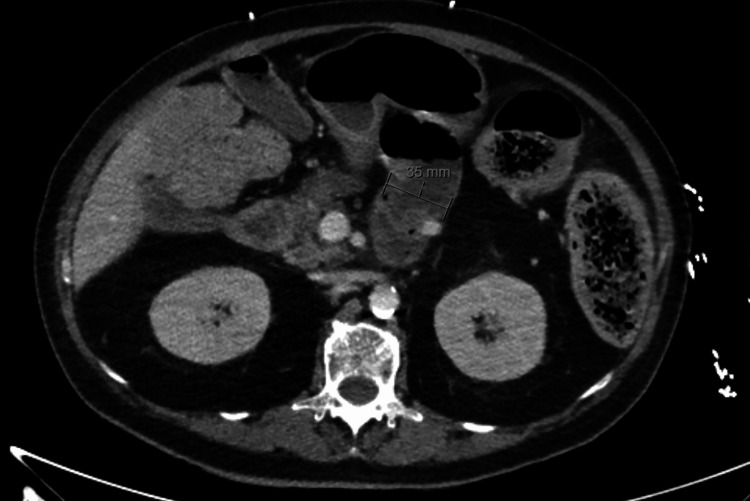
The stomach and proximal jejunum are mildly distended with fluid. A short segment of the small bowel fecalization distal to this suggests slow transit. The fecalized small bowel loop appears to gradually taper and a definite transition point is not identified.

An oliguric acute kidney injury with metabolic acidosis was noted, likely in the context of septic shock. Inflammatory markers were elevated with a C-reactive protein (CRP) of 62 mg/L and a white cell count (WCC) of 25×10^9^/L. CT abdomen revealed an edematous colonic wall with significant fecal loading. Follow-up imaging was consistent with stercoral colitis, displaying marked distension of the rectum and mild distension of the sigmoid and ascending colon with fecal material (Figures 1, 2). Progress imaging over the following weeks showed persistent circumferential wall edema of the descending colon, sigmoid colon, and rectum, indicating persistent colitis, worse than previous studies, despite clearance of fecal impaction.

Along with fluid resuscitation and intravenous antibiotics, a metaraminol infusion was commenced, and she was admitted to the intensive care unit. Given the degree of fecal impaction, she was given aggressive oral aperients plus phosphate fleet enemas. Flexible sigmoidoscopy and washout were performed for assessment with simultaneous decompression and disimpaction of her stercoral colitis. Postoperatively she was commenced on a neostigmine infusion.

Despite extensive attempts at non-operative management over a two-month period and numerous measures taken to mitigate colonic hypomotility, she continued to have recurrent fluctuating pseudo-obstruction with progress abdominal imaging revealing generalized distension of the entirety of the colon and edema of the colonic wall, concerning colitis. Medication management encompassing removal of all opioids, use of aperients including macrogol with electrolytes, lactulose, polyethylene glycol, sodium phosphate "Fleet Enemas," multiple neostigmine infusions, and addition of prokinetics including metoclopramide and erythromycin. In addition, prucalopride was commenced and her electrolytes were optimized with aggressive potassium and magnesium supplementation and the commencement of spironolactone. At times she required 100-150 mmol of potassium supplementation per day. Nevertheless, she continued to have abdominal distension and subsequently had episodes of intractable rectal bleeding, requiring multiple red blood cell transfusions, cumulatively 30 units of packed red blood cells over a four-month period. Multiple progress flexible sigmoidoscopies and decompression were performed which revealed a significant amount of blood pooling within the rectum and circumferential cobblestoning, with a pseudo-membranous appearance. Biopsies taken confirmed features consistent with ischemic colitis. There were no infective pathogens on numerous cultures or biopsies. The patient required four re-admissions to intensive care and repeated neostigmine infusions only had a temporary effect lasting one to two weeks before painless distension recurred. Repeated failures to return to diet prompted the institution of total parenteral nutrition.

The patient remained acutely psychotic and there were significant concerns about any operative management resulting in stoma formation and future ability to self-care. This would mean potentially lifelong dependency, and hence initially, operative intervention was resisted and felt best to be avoided. Multidisciplinary discussions were held regarding this and gastroenterologist input was sought including a second endoscopic opinion.

**Figure 3:**
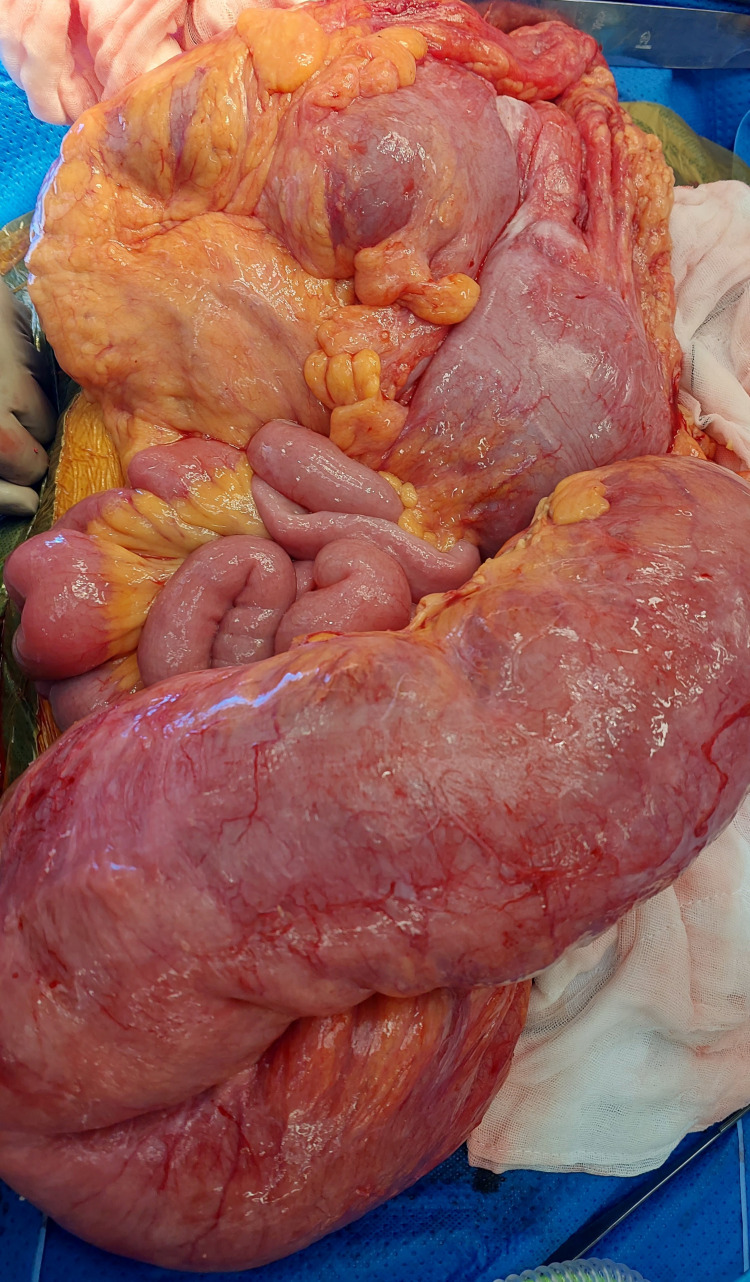
Intraoperative image showing large redundant colon with loss of haustra.

Failure of resolution and further episodes of rectal bleeding had prompted repeat flexible sigmoidoscopy. Unfortunately, she deteriorated rapidly and became febrile, tachycardiac, and hypotensive, requiring metaraminol infusion. She had a sudden drop in hemoglobin to 45 g/L, and an urgent transfusion was commenced. She was intubated and a decision was made for an urgent exploratory laparotomy. Intraoperatively she was found to have a grossly distended colon with a bleeding toxic megacolon. A total colectomy with end ileostomy was performed (Figure 3). Macroscopically, histopathology was consistent with sites of ulceration and a cobbled appearance. This ulceration was present particularly in the distal colon and rectum, with the granulation tissue extending into the muscularis propria microscopically. Between these areas of ulceration, the intact mucosa was consistent with crypt architectural distortion, capillary ectasia, and inflammatory foci. No additional pathology or causative component was found. No histological changes were able to be identified in the enteric nervous system. Immunostaining for cytomegalovirus (CMV) was negative and there was no evidence of active inflammatory bowel disease.

Postcolectomy her abdominal pain and distension significantly improved. Tachycardia improved, as did her inflammatory markers. Her antipsychotic regime was reviewed by psychiatry and risperidone (initially commenced shortly before colectomy) dosage was up-titrated. She continued to have ongoing catatonic features with a partial response to medications and thus involuntary electroconvulsive therapy (ECT) was pursued. Following guardian approval, she underwent 10 sessions and underwent a period of inpatient rehabilitation. She was commenced on high-dose clozapine with symptoms improvement, before being discharged back to her inpatient mental health facility.

## Discussion

Although clozapine has its therapeutic effect by binding to dopamine receptors, its action on multiple other receptors means that it carries a series of adverse effects, including those affecting colonic motility. It is known to have an anticholinergic effect, decreasing peristalsis [3]. Its action on muscarinic receptors directly impedes propulsion motility and defecation. Blocking or inactivating these receptors leads to dilation, colonic distension, and ultimately prolonged transit. Antagonism of serotonin receptors also plays a role in reducing gastrocolic reflexes, increasing colonic compliance, and potentially reducing sensitivity to distension [4]. Ultimately the anticholinergic and antiserotonergic effects of clozapine mean that it decreases gastrointestinal motility. The subsequent hypomotility can lead to obstruction, paralytic ileum, and dilatation. The resultant impaction and colitis can also eventuate in inflammation, impeding the metabolism of clozapine with subsequent increased plasma concentrations of the drug reinforcing all these effects. As the colonic mucosa compresses it is at risk of ischemia and colonic necrosis. The antiadrenergic effects of clozapine can further exacerbate mucosal ischemia due to gastrointestinal hypoperfusion [5]. Furthermore, colonic obstruction and dilatation increase the propensity for bacterial translocation, and where present, endotoxins perpetuate disruption of mucosal tight junctions facilitating further translocation, increasing systemic bacterial load and overall risk of intraabdominal sepsis [6]. Significant morbidity can also arise from aspiration as these patients are prone to inhalation of feculent vomitus and dysphagia [7]. The resultant chemical pneumonitis, pneumonia, or even asphyxiation can cause further morbidity and mortality.

Bowel motility studies suggest that hypomotility occurs in at least 75% of those administered clozapine [8]. A study has demonstrated that 80% of patients on clozapine will have colonic hypomotility, with a median transit time four times longer than those on other antipsychotics. Death in the United Kingdom averages 15 per year, and an Australian and New Zealand study reported a case fatality rate of 18% as a result of adverse clozapine-related gastrointestinal side effects [9]. Internationally, the effects of constipation are now implicated in more clozapine-related deaths than agranulocytosis. The mortality rate of acute fecal impaction or pseudo-obstruction is particularly high in the case of increasing intraluminal pressure with ischemic necrosis and perforation. It is estimated to be 40% if perforation occurs [10]. Instances of clozapine chronically compromising the integrity of the gastrointestinal mucosa are less recognized. Particularly of note in our case was the refractory nature of her condition despite extensive attempts at non-operative management, which was pursued at length due to her social and mental health circumstances. In the above case, eventual rapid deterioration with ongoing lower gastrointestinal bleeding, hemodynamic instability, and a significant hemoglobin drop left no alternative, with hemorrhagic shock and impending perforation prompting the urgency to perform a total colectomy.

Unfortunately, severe clozapine-related gastrointestinal adverse effects can often present in an insidious manner, where there are logistical difficulties in obtaining diagnostic certainty. There are difficulties with gathering sufficient history and collateral from individuals diagnosed with schizophrenia. There may be episodes of diarrhea, hematochezia, and abdominal pain to suggest ischemic necrosis of the colonic wall but ultimately diagnosis is often made in conjunction with findings on CT and colonoscopy. Imaging is useful in demonstrating cecal diameter, which can be associated with the degree of ischemia or even perforation in cases of acute colonic pseudo-obstruction. Pooled data from 400 patients has demonstrated that if the diameter is greater than 14 cm there is a 23% risk of perforation, whereas when the diameter is between 12 and 14 cm the risk is 7% [11]. A delay in diagnosis may mean that the changes are irreversible, highlighting the importance of closely monitoring for the adverse effects of clozapine during its prescription. Given the anticholinergic and antiserotonergic effects of clozapine, caution needs to be made when simultaneously prescribing other medications that have an anticholinergic effect. Close monitoring of bowel habits and clozapine drug levels is important as the risk of hypomotility is often able to be detected clinically and is associated with high plasma clozapine concentrations. Laxatives can be prophylactically commenced and optimization of dietary measures as well as fluid intake can further mitigate the possibility of reduced colonic motility. With any abdominal pain, distension, or signs of sepsis there should be a low threshold for urgent intervention.

The decision for operative management is challenging given the risks involved. A population study evaluating clozapine-associated colonic hypomotility has demonstrated that colonoscopy and surgery had complication rates of 74.6% and a mortality rate of 14.8%, while with surgery alone these rates are 60% and 12.3%, respectively [12]. This study compares such an approach with medical management, which carries a significantly lower complication rate of 44% [12]. As explored in this study, medical management involves fluid resuscitation, optimization of serum electrolyte abnormalities, and minimization of narcotics as well as anticholinergic medications. Neostigmine is effective as an anticholinesterase drug promoting contractility and colonic transit. It can present logistic difficulties as it often needs to be administered in a monitored setting due to the risk of bradycardia. When neostigmine becomes ineffective or is contraindicated, endoscopic decompression can be considered. Endoscopic decompression can be repeated but in instances of colonic ischemia or perforation, or colonic pseudo-obstruction refractory to pharmacological or endoscopic therapies, operative treatment is indicated. A UK surveillance program revealed that surgery was the outcome noted most frequently, inclusive of not only total colectomy (7 of 92) but also hemicolectomy (2 of 92) and proctosigmoidectomy (2 of 92) [10]. Although colectomy offers the definitive treatment of colonic hypomotility, it carries its own morbidity and mortality, and such a decision renders an individual with an already significant mental health burden the responsibility of stoma management, as was the situation in the present case. Surgical reversal and return to gastrointestinal continuity may be fraught given the uncertainty of remaining colonic function, with possible irreversible hypomotility still impacting residual colon or rectum. Again, prevention is better than intervention, and thus, it is imperative to monitor the bowel function of individuals prescribed clozapine to avoid potential life-changing adverse outcomes.

## Conclusions

The anticholinergic effects of clozapine are widespread throughout the gastrointestinal tract, including the colon. It can lead to not only colonic pseudo-obstruction but also stercoral colitis as well as toxic megacolon. In the setting of refractory pseudo-obstruction, progress to surgery may be inevitable and lifesaving. The prescription of clozapine needs to be closely monitored for its adverse effects, e.g., gastrointestinal hypomotility. Without cautious observation, these effects on the colon can be irreversible.
